# Do risk factors differentiate DSM-5 and drive for thinness severity groups for anorexia nervosa?

**DOI:** 10.1186/s40337-024-00966-5

**Published:** 2024-01-11

**Authors:** An Binh Dang, Litza Kiropoulos, Marija Anderluh, David Collier, Fernando Fernandez-Aranda, Andreas Karwautz, Janet Treasure, Gudrun Wagner, Isabel Krug

**Affiliations:** 1 Melbourne School of Psychological Sciences, The University of Melbourne, Redmond Barry Building, Level 7, Room 707, Melbourne, VIC Australia; 2 University Children’s Hospital, University Medical Center Ljubljana SI, Ljubljana, Slovenia; 3 Eating Disorders Unit and SGDP Research Centre, Institute of Psychiatry, King’s College, London, UK; 4 Department of Psychiatry, University Hospital of Bellvitge- IDIBELL, Barcelona, Spain; 5 Department of Child and Adolescent Psychiatry, Medical University of Vienna, Vienna, Austria; 6 Section of Eating Disorders, Department of Psychological Medicine, Institute of Psychiatry, Psychology and Neuroscience, King’s College London, London, UK

**Keywords:** Anorexia nervosa, Risk factors, Severity rating, Drive for thinness, And body mass index

## Abstract

**Background:**

The current study examined whether risk factors for anorexia nervosa (AN) were related to different levels of severity based on (a) the DSM-5/body mass index (BMI) and (b) drive for thinness (DT) severity ratings.

**Methods:**

The sample comprised 153 pairs of individuals with a lifetime diagnosis AN per DSM-IV criteria and their non-ED sisters (*N* = 306, mean age = 26.53; mean current BMI = 20.42 kg/m^2^). The Oxford risk factor interview was used to establish AN-related risk factors. Individuals were categorised into the DSM-5 severity groups based on their lowest BMI, while the DT subscale from the eating disorder inventory-2 was used to classify individuals with AN into low and high DT groups.

**Results:**

Multinominal regression models showed similar risk factors (e.g., perfectionism, having a history of being teased about weight and shape) contributed to the development of AN using the DSM-5 and DT severity ratings. Follow-up analyses across the severity groups for both indices revealed that only childhood perfectionism was found to be more common in the extreme severe DSM-5 BMI severity group compared to the severe DSM-5 group.

**Conclusion:**

Overall, this study found little evidence for AN risk factors being related to the DSM-5 and DT severity ratings. However, given the novelty of this study, replication of the current results is warranted.

**Supplementary Information:**

The online version contains supplementary material available at 10.1186/s40337-024-00966-5.

## Abstract (plain language summary)

Several risk factors, such as childhood obesity, have been found to contribute to the development of Anorexia Nervosa (AN). Yet, we are unsure if there is a set of risk factors that influence different levels of AN severity. While the DSM-5 suggests using BMI to measure severity, recent support favour the usage of drive for thinness (DT) as an alternative severity measure. Therefore, this study aimed to explore risk factors specifically associated with the development of different AN severity levels using both the DSM-5 BMI and DT severity classification systems. We recruited 153 pairs of individuals with a lifetime diagnosis AN per DSM-IV criteria and their non-ED sisters. The Oxford risk factor interview was used to establish AN-related risk factors. We found childhood perfectionism, weight/shape teasing, childhood obesity, and breast-related embarrassment to be significant risk factors for AN. Additionally, childhood perfectionism was more common in the extreme severe DSM-5 group compared to the severe DSM-5 group. This suggests that adding perfectionism-related aspects to prevention and early intervention programs for AN may be beneficial. Considering the novelty of this study, replication of the current results is needed.

**Supplementary Information:**

The online version contains supplementary material available at 10.1186/s40337-024-00966-5.

## Introduction

Risk factors ranging from genetic to psychological factors have been implicated in the aetiology of anorexia nervosa (AN) to inform prevention and treatment programs [1, 2]. To aid the clinical management of AN, the fifth edition of the Diagnostic and Statistical Manual (DSM-5) of Mental Disorders [3] presented a new severity rating for AN, which categorises individuals with AN into the mild, moderate, severe, and extreme severe severity groups based-on body-mass index (BMI). However, studies have found the clinical usefulness of the DSM-5 severity rating for AN to be limited [4, 5]. As such, researchers have explored alternative severity classifications for AN, including transdiagnostic indices (e.g., drive for thinness [DT]) and weight and shape concerns), and found these alternative severity ratings to be superior in indexing eating disorder (ED) psychopathology in comparisons to the DSM-5 BMI severity rating [4]. Despite many studies having been conducted on both AN risk factors [6] and severity ratings [4], researchers have yet to examine risk factors specifically associated with the development of different AN severity levels.

### Risk factors for anorexia nervosa

Several risk factors have been implicated in the aetiology of AN, which include amongst others a family history of an ED [7, 8] or traits such as perfectionism, low self-esteem, or obsessive personality traits [8, 9]. However, most of these studies used self-report assessments, and are thus limited by recall biases [10]. The Oxford Risk Factor Interview (ORFI) [11], a semi-structured interview, has been considered the gold standard ED risk factor assessment tool. This is due to the ORFI’s ability to assist participants in recalling information related to the autobiographic anchor point and the use of probes to encourage detailed descriptions of risk factor-related events (e.g., parental separation).

Most studies using the ORFI employed case–control designs, which compared individuals with AN to individuals with no DSM-5 ED diagnosis or no psychiatric disorders [6, 12]. These studies [6, 12] mainly found, similar to the studies using self-report measures [8], that perfectionism, negative self-evaluation, weight and shape concerns, and familial history of AN were risk factors associated with the development of AN. However, these ORFI case–control studies are limited by their inability to control for environmental and family factors that might contribute to the development of AN [13].

To overcome these limitations, studies have started employing samples of sister pairs discordant for AN, i.e., individuals with AN and their non-ED sisters. This design allows for control of cultural, environmental, and family factors so that individual-specific (i.e., non-shared) factors can be more distinctively assessed [14, 15]. Karwautz et al. [14, 15], employing this discordant sister pair design with 90 and 256 participants, respectively, found that individuals with AN, compared to their non-ED sisters, experienced higher rates of weight and shape teasing, exposure to critical comments, and disruptive life events. However, further research is required to elucidate the relationship between varying severity levels of AN and the associated risk factors of AN.

### Combining AN risk factors and severity

While the medical literature has extensively investigated the relationships between risk factors and symptom severity in conditions like coronavirus (COVID-19) disease [16] and acute stroke [17], such exploration has been lacking in psychiatric research for conditions such as AN. For example, having a history of obesity and diabetes has been found to increase the risk of having severe COVID-19 (e.g., respiratory failure, intensive care unit admission [16] This means that the medical field has gained a certain understanding of how certain risk factors not only increase the risk of having a disease but also contribute to progressing to a greater severity (e.g., impairments, mortality). Using such information, medical professionals can proactively identify patients at a high risk of developing severe illnesses, which in turn allows them to inform medical decisions such as the need for hospitalisation or treatment frequency. However, in the ED literature, such a severity-risk factor approach has not yet been assessed.

### DSM-5 severity rating for AN based on BMI

To date, the most well-known and widely used severity classification system for AN was proposed by the DSM-5 using BMI [4]. This DSM-5 severity rating categorises individuals with AN into “mild” (≥ 17.0 kg/m^2^), “moderate” (16–16.99 kg/m^2^), “severe” (15–15.99 kg/m^2^) and “extreme” (< 15 kg/m^2^) severity groups. However, the clinical utility of the BMI specifiers for AN has consistently been questioned by recent research [4, 5]. Dang et al. [4] conducted a meta-analysis (*N* = 22) assessing all the current DSM-5 severity rating studies for AN, bulimia nervosa (BN) and binge eating disorder (BED). In relation to AN, which comprised five studies [18–22], the review did not find significant differences in ED psychopathology across the DSM-5 BMI severity groups. This means that although BMI has been widely utilised in both clinical practice and empirical research to index severity [23], the level of evidence regarding the DSM-5 AN severity rating is still unknown. If the DSM-5 severity rating is valid, besides indexing the intensity of ED psychopathology, it should also be able to distinguish other clinical information such as the prognosis or factors involved with the development (i.e., risk factors) and maintenance of AN.

#### Drive for thinness as an alternative severity rating for AN

Given the ambiguity surrounding the DSM-5 AN severity rating, researchers have explored alternative severity classifications for AN such as overvaluation of weight and shape [5, 22], and DT [4, 24]. Studies have consistently found that individuals with AN who scored high on DT had more severe attitudinal and behavioural ED symptoms, as well as more psychiatric comorbidity compared to those with low DT [25, 26]. DT has been considered as another promising alternative severity rating for EDs in general and AN specifically. Krug et al. [24] found that DT provided more significant differences than the DSM-5 BMI severity groups in ED and general psychopathology, with the high DT group scoring higher on these variables than the low DT individuals. Given that previous research has only assessed the clinical utility of DT severity for AN on ED and general psychopathology [4], it is important to expand the scope of severity rating research to look at the relationship between AN-related risk factors and DT as an alternative severity indicator for AN.

### The current study

In this study, by using a sister-pair discordant design (comprising individuals with a lifetime DSM-IV diagnosis of AN and a sister without an ED diagnosis), we aimed to identify specific risk factors linked to varying levels of AN severity according to either DSM-5 or DT severity rating. This extends the work of Karwautz et al. [15], who employed a similar design (and part of an overlapping sample) but focused on identifying general risk factors for AN development without delving into specific risk factors contributing to different AN severity levels per DSM-5 and DT ratings. To do so, we first assessed which ORFI risk factors were related to the development of AN based on both the DSM-5 BMI and DT severity ratings, while using non-ED sisters as controls to enhance the distinctiveness of individual factors. We then focused only on the significant risk factors from these primary analyses and examined whether there were significant differences in the frequency of the exposure of each of these significant risk factors among the DSM-5 BMI (mild/moderate, severe, and extreme severe) and DT (high vs. low) severity groups. Understanding such relationships would not only provide insights into the clinical utility of these two severity classification systems, but would also provide a guide for developing more proactive prevention programs and informing public clinical management policies for AN.

## Method

### Design

This study used a discordant sister pair design, which meant that women who met the diagnosis of AN according to DSM-IV criteria [27] were compared to their non-ED sisters. Individuals meeting the criteria for AN in the DSM-IV would generally still qualify for an AN diagnosis under the DSM-5 criteria [28]. This is because the DSM-5 broadens the AN criteria, removing specific weight thresholds and the amenorrhea requirement to encompass a wider spectrum of individuals with the disorder. This approach aligns with previous studies in this field [20]. The data for this study were collected as part of a multicentre European project.

### Recruitment procedure

Four different sites specialising in EDs participated in this study: the Institute of Psychiatry, London, UK; the University of Vienna, Austria; the University Hospital of Bellvigte, Barcelona, Spain, and the University Children’s Hospital, Ljubljana, Slovenia. Between 1999 to 2002, participants were recruited from both clinical settings and community resources such as websites and volunteer databases from previous research studies (for more information please refer to Giles et al. [27].

### Participants

The current study comprised 153 pairs of individuals with AN and their non-ED sisters (*N* = 306). Their combined mean age was 26.25 (*SD* = 8.00), and their mean current BMI was 20.42 kg/m^2^ (*SD* = 3.60). Most participants were employed (43.9%). Among the individuals with AN, the mean age of illness onset and duration of illness was 16.98 years (*SD* = 4.92) and 6.51 years (SD = 6.59) respectively.

Inclusion criteria for individuals with AN were: (1) female gender; (2) a lifetime diagnosis of AN according to DSM-IV criteria derived from the Longitudinal Interval Follow-up Evaluation [LIFE] interview [29]; and (3) having a sister close in age. The exclusion criteria for both individuals with AN and their non-ED sisters included not having a current psychotic disorder and learning disability.

Non-ED sisters were included in the study if they did not have a diagnosis of an ED which was screened by the LIFE interview [29]. To control for environmental factors, the sister pairs needed to have an age gap of less than 10 years and must have lived together in the same family for at least 8 years. If the patient had more than one sister, the sister who was closest in age was included.

### Measure

Clinical psychologists, with over 5 years of research experience in the field of EDs, conducted all interviews outlined below. These psychologists received training from senior ED clinicians based in different European countries. Although inter-rater reliability was not assessed in this study, prior research [12, 30] has consistently shown adequate reliability and validity for the semi-structured interviews (e.g., LIFE [29], ORFI [11]) employed in the current study.

#### Social demographic and clinical information

The first part of the EATATE [31], a semi-structured interview to assess ED symptoms, was used to derive information regarding participants’ age, occupation, current height, current weight, and highest and lowest weight since reaching adulthood. From this information, the current, lowest, and highest BMI was calculated. In addition, current weight and height were measured at the ED units at intake.

#### Longitudinal interval follow-up evaluation (LIFE)

This study used an adapted version of the European LIFE [29] to measure lifetime ED history among participants with AN. This included constructing anchor points and timelines for the development of ED symptoms, symptom severity, and psychiatric treatment received. Non-ED sisters were also screened for any ED and ED-related features using the same LIFE interview This was done to ensure that the non-ED sisters have never met any ED diagnoses. This interview has demonstrated adequate reliability and validity in prior research (e.g., 31).

#### Oxford risk factor interview for eating disorders (ORFI)

The ORFI [11] was used to examine specific risk factors associated with the development of AN. At the beginning of the interview, the clinicians aimed to identify the period before the onset of AN, where the age of onset is defined as the time when the first significant persistent disordered eating pattern began [11, 12, 32]. Drawing from prior research [11, 32], a comprehensive array of potential risk factors was examined and categorised into three overarching domains: individual vulnerability, environmental risk, and dieting vulnerability. Each domain included several subdomains (e.g., childhood characteristics, childhood abuse), representing specific types of exposure within the main domain. For example, to assess negative self-evaluation, participants were asked, “In general, as a child or adolescent, how did you feel about yourself compared to other people? Did you feel that you were the same, better, or worse than the others?” Another example item to assess the risk of deliberate self-harm included “Have you ever tried to hurt yourself without the intention of committing suicide?”. Exposure to a risk factor was rated on a 5-point scale ranging from 0 = *no exposure* to 4 = *high severity, long duration,* or *high frequency of exposure*. The data were then recoded into 0 = *no definite exposure* (initially coded 0, 1, or 2) versus 1 = *definite exposure* to reduce the likelihood of false positives, (initially coded 3 or 4). The ORFI has been found to have good inter-rater reliability with a high level of agreement across the risk factor domains (main weighted kappa: 0.66, SD: ± 0.17) [12]. For more information, please refer to Fairburn et al. [11].

### Eating disorders inventory-2 (EDI-2)

The EDI-2 [33] is a 91-item self-report measure of cognitive and behavioural characteristics frequently associated with EDs. We used the EDI-2 DT subscale as an alternative severity rating for AN. EDI-2 DT subscale includes seven items (rating from 1 = never to 6 = always), which assesses excessive dieting anxiety, preoccupation with weight, and fear of weight gain (e.g., “I am preoccupied with the desire to lose weight”). The DT subscale has demonstrated excellent internal reliability with α = 0.85 [24].

### DSM-5 AN severity groups

This study recruited participants with a lifetime diagnosis of AN according to DSM-IV criteria, meaning that some individuals had already recovered from their ED. Therefore, to have an accurate representation of the level of AN severity that the participants had experienced during their illness, we used the lowest lifetime BMI after reaching 18 years old rather than the current BMI to categorise participants into the four BMI AN severity groups.

Using this minimum BMI severity index, we categorised the AN participants according to the four DSM-5 severity groups. However, since there were only a few individuals who were categorised in the mild and moderate groups [*mild (n* = 10), *moderate* (*n* = 21)], we had to reclassify the mild and the moderate AN DSM-5 severity groups into one “mild/moderate” group. This reclassification has been reported in previous studies [33] to allow a sufficiently large sample size across the groups to undertake meaningful comparisons. Overall, a total of 31 participants were classified as mild/moderate, 40 as severe and 82 as extreme severe using the DSM-5 severity ratings based on the lowest lifetime BMI.

### Drive for thinness severity groups

The alternative DT severity rating system was derived from the EDI-2 [33] DT subscale. The DT subscale score was derived by summing the scores of all seven DT items. For screening purposes, Garner [34] recommended a cut-off score of > 14 to differentiate those with high DT from those with low DT, which subsequently has been applied across various research studies (e.g., 24). Using the same cut-off of 14, we classified individuals with AN into low DT (*n* = 60) and high DT (*n* = 69) individuals.

### Statistical analysis

Analyses of variance (ANOVA) and chi-square analysis were used to compare sociodemographic and clinically related information between the non-ED sisters and the two AN severity indices (DSM-5 BMI and DT) for continuous and categorical variables respectively. Eta-squared coefficient (η^2^) as the measurement of effect size for ANOVA (values of 0.06, 0.10, and 0.25 were interpreted as low-poor, moderate-medium, and large-high effect sizes respectively; 36). Cramer’s V coefficient was used as a measurement of effect size for chi-square analyses (values of 0.06; 0.15, and 0.30 were interpreted as low-poor, moderate-medium, and large-high effect sizes respectively [35]). Each risk factor was coded as 0 for “no – the risk was not presented”, and 1 for “yes – the risk was present”.

Multinominal logistic regression analyses with “non-ED sisters” as the reference group were employed to investigate which risk factors were significantly associated with higher odds of developing the different AN severity categories based on the DSM-5 BMI and DT severity ratings. To account for multiple testing, we applied the Benjamini and Hochberg correction for multiple comparison method, where *p*-values were ranked, and then adjusted by multiplying each by a factor of m/k, with m representing the number of independent tests and k being the position of a *p*-value in the sorted array [36]. The chosen alpha level was 0.05. The results of the significant tests (*p*-value) and the effect-size estimates were utilised to evaluate the validity of both the DSM-5 BMI and the alternative DT severity ratings for AN.

The odds ratio (OR) was used as the measurement of the effects of being exposed to AN-related risk factors and the development of different levels of AN severity based on the DSM-5 BMI and DT severity ratings. An OR of greater than 1.00 indicates that exposure was associated with higher odds of developing AN. With an OR of less than 1.0 is associated with lower odds of developing AN. Following Chen’s [37] rules-of-thumb OR of 1.68, 3.47, and 6.71 are considered as small, medium, and large effect-size respectively. Chi-square tests were conducted to test for the differences in the rate of being exposed to each significant risk factor for the DSM-5 BMI and DT severity groups.

## Result

### Sociodemographic and clinical-related information

The sociodemographic and clinical-related information is displayed in Table 1. As expected, there were significant differences in lowest (*p* < 0.001), current (*p* < 0.001), and highest (*p* < 0.001) BMI between individuals with AN and their non-ED sisters. There were no significant differences in age between non-ED sisters and individuals with AN. Further information on sociodemographic of the sample can be found in Additional file 1: Table S1.

**Table 1:** Social and clinical related information of non-ED sisters and individuals with AN

		Comparisons between non-ED sisters and individuals with AN			Comparisons between DSM-5 severity groups				Comparisons between Drive for thinness (DT) severity groups		
	Total Mean (SD)	Non-ED sisters	Individuals with AN	*P* values (η^2^)	Mild/Moderate	Severe	Extreme Severe	*P* values (η^2^)	Low DT	High DT	*P* values (η^2^)
Clinical information											
Age (yrs-old)	26.25 (8.00)	26.55 (8.30)	25.97 (7.73)	0.516 (0.001)	26.72 (6.67)	25.76 (6.05)	25.78 (8.81)	0.826 (0.003)	25.19 (6.72)	26.30 (7.78)	0.156 (0.008)
Onset ED (yrs-old)	16.98 (4.92)	–	16.98 (4.92)	–	17.71 (5.01)	17.41 (4.18)	15.82 (3.36)	**0.029 (0.046)**	16.52 (4.30)	16.50 (3.82)	0.713 (0.001)
Duration ED (yrs)	6.51 (6.59)	–	6.51 (6.59)	–	6.21 (6.86)	5.36 (3.97)	7.21 (7.11)	0.314 (0.015)	6.13 (6.07)	6.93 (6.11)	0.465 (0.004)
Current BMI (kg/m^2^)	20.42 (3.60)	22.08 (3.90)	18.74 (2.41)	** < 0.001 (0.217)**	19.30 (1.52)	19.30 (2.30)	18.31 (2.68)	0.063 (0.036)	18.63 (2.34)	19.07 (2.47)	0.078 (0.013)
Lowest BMI (kg/m^2^)	17.11 (3.61)	19.81 (2.82)	14.48 (2.00)	** < 0.001 (0.547)**	17.04 (1.17)	15.45 (0.30)	13.04 (1.36)	** < 0.001 (0.734)**	14.47 (2.18)	14.50 (1.81)	0.933 (0.001)
Highest BMI (kg/m^2^)	22.58 (4.19)	23.58 (4.78)	21.56 (3.20)	** < 0.001 (0.059)**	22.22 (2.80)	21.17 (3.65)	21.02 (3.03)	0.082 (0.033)	21.12 (3.18)	21.62 (3.18)	0.075 (0.013)
Social information				*P* values (V)				*P* values (V)			*P* values (V)

Bold significant comparison using adjusted p-value using the Benjamini‐Hochberg false discovery rate procedure (.05 level)

Using the DSM-5 BMI severity rating for AN, significant differences between the BMI severity groups were found for the age of illness onset (*p* = 0.029). However, post-hoc comparisons did not reveal where the differences between the groups lay (Table 1). No other significant differences emerged in the sociodemographic and clinically related information between the DSM-5 BMI severity groups. For the DT groups, no significant differences in any of the sociodemographic (Additional file 1: Table S1) and clinically related information were observed.

### Exposure to AN-related risk factors and odds of being in the DSM-5 severity groups

Using non-ED sisters as a reference group, multinomial logistic regression models (Table 2) showed that the following factors increase the risk of developing an AN diagnosis across the DSM-5 BMI severity groups: childhood perfectionism (mild/moderate, OR = 6.67; severe, OR = 3.13; extreme severe, OR = 10.00); childhood obesity (mild/moderate, OR = 25.00; severe, OR = 20.00; extreme severe, OR = 16.67); having a history of being teased about weight, shape, or appearance (mild/moderate, OR = 4.50; severe, OR = 3.40; extreme severe, OR = 3.86); and having a history of feeling embarrassment about one’s breasts (mild/moderate, OR = 7.14; severe, OR = 5.00; extreme severe, OR = 4.35).

**Table 2:** Distribution of risk factors for across the mild/moderate, the severe and the extreme severe AN DSM-5/BMI severity groups, and high and low drive for thinness groups results using multinominal logistic regression, with non-ED sisters as the reference group

Severity groups	DSM-5 Mild/Moderate		DSM-5 Severe		DSM-5 Extreme		Low DT		High DT	
	OR [95% CI]	*p*	OR [95% CI]	*p*	OR [95% CI]	*p*	OR [95% CI]	*p*	OR [95% CI]	*p*
Individual vulnerability domain										
*Subdomain 1 (childhood characteristic)*										
Negative self-evaluation	0.58 [0.26; 1.35]	0.455	1.15 [0.55; 2.44]	0.885	1.10 [0.59; 2.04]	0.890	0.62 [0.31; 1.22]	0.415	1.69 [0.84; 3.45]	0.391
Perfectionism	6.67 [2.33; 20.00]	**0.006**	3.13 [1.20; 8.33]	**0.003**	10.00 [4.55; 20.00]	< **0.001**	8.26 [3.57; 19.23]	< **0.001**	6.67 [2.86; 14.29]	< **0.0001**
Shyness	1.96 [0.83; 4.76]	0.372	1.15 [0.53; 2.50]	0.906	0.91 [0.47; 1.79]	0.897	0.90 [0.43; 1.87]	0.894	1.39 [0.69; 2.86]	0.631
Extreme compliance	1.05 [0.42; 2.63]	0.952	1.89 [0.87; 4.00]	0.366	1.52 [0.79; 2.86]	0.451	1.27 [0.62; 2.60]	0.771	1.75 [0.87; 3.57]	0.359
No close friends	0.42 [0.13; 1.37]	0.407	1.01 [0.43; 2.38]	0.978	0.46 [0.21; 1.02]	0.232	1.03 [0.46; 2.32]	0.951	0.38 [0.15; 0.93]	0.169
No opposite gender friends	0.43 [0.15; 1.25]	0.365	0.55 [0.23; 1.30]	0.416	1.10 [0.55; 2.17]	0.895	0.67 [0.30; 1.49]	0.610	0.78 [0.36; 1.69]	0.773
School absence through anxiety	–	–	0.11 [0.01; 0.88]	0.178	0.14 [0.03; 0.66]	0.089	–	–	0.27 [0.07; 1.00]	0.216
*Subdomain 2 (premorbid psychiatric disorder)*										
Major depression	1.89 [0.75; 4.76]	0.419	2.94 [1.35; 6.67]	0.051	2.00 [1.05; 3.85]	0.180	2.04 [0.97; 4.17]	0.232	2.50 [1.22; 5.00]	0.087
Anxiety disorders	1.23 [0.51; 3.03]	0.839	2.04 [0.96; 4.35]	0.239	1.47 [0.80; 2.63]	0.444	1.19 [0.60; 2.38]	0.827	1.96 [1.01; 3.85]	0.207
Substance abuse	0.65 [0.07; 6.25]	0.889	0.48 [0.55; 4.55]	0.774	1.23 [0.31; 5.00]	0.894	0.74 [0.07; 7.14]	0.883	3.33 [0.75; 14.29]	0.361
Soft drug abuse	3.57 [1.43; 9.09]	0.057	2.13 [0.85; 5.26]	0.368	1.23 [0.55; 2.78]	0.827	0.88 [0.34; 2.27]	0.884	2.13 [0.94; 4.76]	0.249
*Subdomain 3 (behavioral problems)*										
Marked conduct problem	1.11 [0.80; 100]	0.263	2.94 [0.15; 50.00]	0.734	1.59 [0.09; 25.00]	0.893	–	–	5.56 [0.49; 50.00]	0.411
School absence (truancy)	0.72 [0.10; 5.26]	0.889	1.41 [0.32; 6.25]	0.846	1.23 [0.38; 4.00]	0.897	0.64 [0.13; 3.13]	0.813	1.67 [0.51; 5.56]	0.678
Deliberate self-harm	7.14 [1.75; 25.00]	0.053	5.56 [1.39; 20.00]	0.097	4.17 [1.22; 14.29]	0.127	4.17 [0.97; 16.67]	0.233	8.33 [2.08; 33.33]	**0.030**
*Subdomain 4 (physical health)*										
Severe personal health problems	2.22 [0.83; 5.88]	0.363	1.37 [0.51; 3.57]	0.781	1.33 [0.62; 2.94]	0.718	1.56 [0.68; 3.70]	0.558	2.00 [0.85; 4.55]	0.362
Childhood obesity	25.00 [7.14; 100.00]	< **0**.**001**	20.00 [5.88; 100.00]	** < 0.001**	16.67 [4.76; 50.00]	< **0.001**	25.00 [5.26; 100.00]	**0.001**	33.33 [7.69; 100.00]	< **0.001**
*Subdomain 5 (parental psychiatric disorder)*										
Parental depression	1.14 [0.40; 3.23]	0.877	0.68 [0.23; 2.00]	0.733	1.38 [0.68; 2.78]	0.650	1.82 [0.83; 4.00]	0.377	0.82 [0.34; 2.00]	0.849
Parental anxiety	0.56 [0.06; 4.76]	0.811	2.38 [0.65; 8.33]	0.435	2.08 [0.75; 5.88]	0.419	0.82 [0.20; 3.45]	0.891	2.33 [0.70; 7.69]	0.418
Parental drug abuse	1.69 [0.55; 5.26]	0.635	0.75 [0.20; 2.86]	0.853	1.03 [0.43; 2.50]	0.961	0.71 [0.25; 2.00]	0.775	1.23 [0.47; 3.23]	0.848
Environmental Risk Domain										
*Subdomain 1 (parental problem)*										
Distress due to low parental contact	0.76 [0.15; 3.85]	0.892	1.18 [0.35; 3.85]	0.886	1.82 [0.72; 4.55]	0.462	0.65 [0.19; 2.27]	0.749	1.55 [0.58; 4.15]	0.651
Distress due to separation from parents	0.84 [0.16; 4.55]	0.890	0.59 [0.11; 3.23]	0.782	0.43 [0.12; 1.56]	0.447	0.35 [0.07; 1.81]	0.442	0.57 [0.14; 2.36]	0.713
Distress due to parental arguments	2.13 [0.92; 5.00]	0.284	0.99 [0.44; 2.22]	0.984	2.63 [1.47; 4.76]	0.142	3.31 [1.70; 6.45]	0.006	1.08 [0.55; 2.12]	0.877
Parental criticisms	1.54 [0.55; 4.35]	0.682	1.03 [0.38; 2.78]	0.968	1.45 [0.69; 3.03]	0.609	1.11 [0.47; 2.60]	0.887	1.56 [0.69; 3.56]	0.558
Parental high expectation	1.39 [0.59; 3.23]	0.724	0.74 [0.33; 1.64]	0.726	1.53 [0.85; 2.78]	0.419	1.89 [0.97 3.70]	0.239	0.92 [0.46; 1.82]	0.885
Parental over-involvement	0.88 [0.33; 2.38]	0.880	1.18 [0.46; 3.03]	0.902	0.84 [0.41; 1.72]	0.837	0.74 [0.32; 1.70]	0.731	1.21 [0.56; 2.63]	0.836
Parental neglect	0.31 [0.03; 2.86]	0.564	4.35 [1.37; 14.29]	0.092	0.56 [0.17; 1.92]	0.639	0.69 [0.18; 2.60]	0.813	1.77 [0.63; 5.00]	0.547
Parental affection	0.75 [0.29; 1.96]	0.790	1.39 [0.58; 3.33]	0.719	0.70 [0.35; 1.37]	0.528	0.76 [0.36; 1.62]	0.479	1.32 [0.63; 2.75]	0.730
*Subdomain 2 (disruptive events)*										
Distress due to parental death	3.33 [0.51; 20.00]	0.451	2.00 [0.30; 12.50]	0.733	1.27 [0.20; 7.69]	0.886	0.73 [0.07; 7.41]	0.889	3.68 [0.81; 16.67]	0.314
Distress due to parental chronic illness	1.20 [0.35; 4.00]	0.894	2.13 [0,81; 5.56]	0.375	0.95 [0.38; 2.44]	0.947	1.17 [0.44; 3.11]	0.892	1.51 [0.60; 3.77]	0.655
Distress due to frequent house moves	2.00 [0.37; 11.11]	0.691	3.13 [0.78; 12.50]	0.364	3.33 [1.04; 10.00]	0.193	2.47 [0.68; 8.93]	0.422	2.29 [0.62; 8.47]	0.444
*Subdomain 3 (teasing and bullying)*										
Teasing (not concerning weight, shape, eating or appearance)	0.84 [0.23; 3.06]	0.894	1.37 [0.50; 3.75]	0.779	2.29 [1.12; 4.67]	0.123	1.27 [0.53; 3.04]	0.815	1.88 [0.83; 4.27]	0.372
Bullying	0.24 [0.05; 1.04]	0.237	0.27 [0.08; 0.92]	0.181	0.38 [0.17; 0.85]	0.113	0.35 [0.14; 0.89]	0.147	0.22 [0.07; 0.66]	0.055
*Subdomain 4 (sexual and physical abuse)*										
Sexual abuse	1.31 [0.53; 3.25]	0.795	2.39 [1.13; 5.08]	0.130	1.34 [0.72; 2.51]	0.633	1.41 [0.70; 2.82]	0.618	1.36 [0.68; 2.72]	0.653
Physical abuse	0.57 [0.18; 1.79]	0.614	1.22 [0.53; 2.82]	0.842	0.92 [0.46; 1.84]	0.883	0.72 [0.32; 1.63]	0.707	1.35 [0.65; 2.79]	0.686
Dieting Vulnerability Domain										
*Subdomain 1 (dieting risk)*										
Influenced by parent eating disorders	1.63 [0.27; 9.71]	0.810	1.35 [0.24; 7.69]	0.890	0.30 [0.03; 2.81]	0.562	1.13 [0.23; 5.46]	0.924	0.84 [0.15; 4.69]	0.889
Family member dieting for shape and weight	1.96 [0.76; 5.03]	0.419	1.05 [0.43; 2.58]	0.953	0.89 [0.42; 1.87]	0.895	0.91 [0.40; 2.07]	0.873	1.15 [0.53; 2.50]	0.893
Critical comments by family about weight, shape or eating	1.05 [0.45; 2.46]	0.947	1.77 [0.84; 3.76]	0.373	1.19 [0.65; 2.15]	0.811	1.16 [0.59; 2.27]	0.857	1.35 [0.70; 2.62]	0.644
Repeated comments by others about shape and weight	0.75 [0.24; 2.33]	0.832	1.74 [0.73; 4.17]	0.446	1.56 [0.76; 3.19]	0.462	1.18 [0.51; 2.74]	0.886	1.61 [0.73; 3.53]	0.472
Teasing about shape, weight, or appearance	4.50 [1.86; 10.99]	0.**010**	3.40 [1.48; 7.81]	**0.038**	3.86 [1.95; 7.63]	**0.002**	4.93 [2.40; 10.10]	< **0.001**	3.61 [1.75; 7.46]	**0.007**
Dieting at school common	2.48 [0.76; 8.06]	0.379	2.20 [0.73; 6.58]	0.416	2.04 [0.83; 5.03]	0.361	2.23 [0.83; 5.95]	0.367	1.82 [0.66; 5.05]	0.495
*Subdomain 2 (pudicity domain)*										
Preparation for menarche	0.45 [0.14; 1.43]	0.421	0.86 [0.36; 2.04]	0.895	0.64 [0.31; 1.33]	0.476	0.96 [0.43; 2.15]	0.950	0.57 [0.24; 1.37]	0.453
Menarche mainly unpleasant	2.86 [0.96; 8.33]	0.236	2.04 [0.74; 5.88]	0.416	3.03 [1.32; 6.67]	0.066	3.32 [1.22; 9.01]	0.110	4.90 [1.96; 12.35]	**0.008**
Scared by early breast development	0.29 [0.09; 0.93]	0.182	0.56 [0.23; 1.35]	0.449	0.41 [0.19; 0.85]	0.113	0.30 [0.13; 0.73]	0.061	0.39 [0.17; 0.89]	0.458
Teasing about breast as early age	0.32 [0.10; 1.09]	0.254	0.51 [0.19; 1.32]	0.417	0.22 [0.09; 0.57]	**0.021**	–	–	0.40 [0.15; 1.01]	0.233
Breast as source of embarrassment	7.14 [2.78; 16.67]	**0.001**	5.00 [2.08; 11.11]	**0.004**	4.35 [2.13; 9.09]	**0.001**	3.39 [1.48; 7.75]	**0.037**	5.41 [2.48; 11.76]	**0.001**

Adjusted p-value using the Benjamini‐Hochberg false discovery rate procedure

Bold significant comparison using adjusted p-value using the Benjamini‐Hochberg false discovery rate procedure (.05 level)

Follow-up chi-square analyses were conducted on the risk factors that significantly contributed to the development of AN based on the DSM-5 BMI severity classification (Table 3). Among all the significant risk factors, significant differences between the DSM-5 BMI severity groups were only obtained for childhood perfectionism (*p* = 0.039), with individuals in the extreme severe DSM-5 BMI severity group presenting with more childhood perfectionism compared to individuals in the severe DSM-5 BMI severity group. No other significant differences between the DSM-5 BMI severity groups in the rate of being exposed to the remaining significant AN risk factors were found.

**Table 3:** Follow-up chi-square analyses on the risk factors that significantly contributed to the development of AN based on the DSM/BT and DT classification

	DSM-5								DT				
	Mild/moderate (1)		Severe (2)		Extreme (3)		χ^2^ (*p* value; Cramer’s V)	Post-hoc	Low		High		χ^2^ (*p* value; Cramer’s V)
	*N*	%	*n*	%	*n*	%			*n*	%	*n*	%	
Perfectionism	11	17.2	11	17.2	42	65.6	6.86 (0.032; 0.212)	3 > 2 (.039)	31	47	35	53	0.43 (0.515; 0.042)
Childhood obesity	11	25.6	12	27.9	20	46.5	1.45 (0.480; 0.098)	–	15	40.5	22	59.5	1.70 (0.193; 0.083)
Teasing about shape, weight, or appearance	13	23.2	14	25	29	51.8	0.48 (0.787; 0.056)	–	34	50	34	50	0.01 (0.937; 0.005)
Breast as source of embarrassment	4	15.4	9	34.6	13	50	1.30 (0.521; 0.092)	–	22	39.3	34	60.7	3.59 (0.058; 0.121)

### Exposure to AN-related risk factors and odds of being in the high/low DT severity groups

For DT, multinomial logistic regression models (using non-ED sisters as the comparison group to either low or high DT) found childhood perfectionism (low DT, OR = 8.26; high DT, OR = 6.67); childhood obesity (low DT, OR = 25.00; high DT, OR = 33.33); having a history of being teased about weight, shape, or appearance (low DT, OR = 4.93; high DT, OR = 3.61); and feeling embarrassed about breasts (low DT, OR = 3.39; high DT, OR = 5.41) to be significant risk factors for both the low and high DT groups. Conversely, in comparison to non-ED sisters, having a history of self-harm was found to only increase the risk of being assigned to the high DT AN severity group (OR = 8.33), but not the low DT group. Additionally, in comparison to non-ED sisters, those in the low DT group were more likely to feel distress due to parental arguments (OR = 3.31; Table 2).

Follow-up chi-square analyses were conducted on the risk factors that significantly contributed to the development of AN based on the DT classification (Table 3). However, these analyses showed no significant differences between the low and high DT groups in the frequency of exposure to each of these risk factors.

## Discussion

Using a discordant AN sister pair design, the current study identified risk factors that may contribute to the development of different AN severity levels based on the DSM-5 BMI and DT severity classification systems. Several factors (e.g., perfectionism, teasing about weight and shape – see details below) were found to contribute to the development of AN based on both severity indices. However, when looking at the differences in the rate of being exposed to these significant risk factors across the DSM-5 BMI and DT severity groups, besides childhood perfectionism, no other significant risk factor was found.

### Risk factors for AN based on the DSM-5 and DT severity indices

#### Comparisons between non-ED sisters and AN DSM-5 and DT severity groups

Compared to non-ED sisters, we found that almost the same AN risk factors were associated with the severity spectrum of the DSM-5 BMI and DT classification systems. In accordance with the literature, we found that childhood obesity [38, 39], perfectionism [39, 40]; being teased about weight and shape [41] and feeling embarrassed about breasts [42] were associated with having a subsequent diagnosis of AN using these two severity indices.

This overlap in AN risk factors across the DSM-5 BMI and DT severity indices may imply that these two severity classification systems could be correlated with each other. However, in the current study, opposite patterns in the strength of effects across the two severity ratings were found for several risk factors (e.g., childhood obesity). For example, in comparison to non-ED sisters, childhood obesity was associated with a higher risk of being classified into the mild/moderate DSM-5 BMI severity groups. Contrastingly, those reported having childhood obesity were found to have a higher likelihood of being classified into the high DT (as opposed to low DT) severity group.

These patterns of results are somewhat consistent with the literature [43, 44]. Specifically, studies have shown that a higher childhood BMI was directly associated with an elevated adulthood BMI (i.e., mild/moderate DSM-5 BMI severity) and indirectly through both DT in childhood and adulthood (high DT group; 44). This may suggest that different underlying mechanisms might mediate the relationships between AN risk factors and AN severity levels based on these two severity classifications. Further research using longitudinal designs is needed to establish a temporal relationship of the potential different mechanisms that might lead to the different DSM-5 BMI and DT severity groups when compared to a non-ED control sample.

#### Differences across DSM-5 and DT severity groups in the significant risk factors

Across all DSM-5 and DT severity groups, those with an AN diagnosis were more likely to exhibit childhood perfectionism. However, when assessing differences across the severity groups, only those in the extreme DSM-5 BMI severity group were significantly more likely to present with childhood perfectionism compared to those in the DSM-5 severe group. This finding aligns with previous studies [45, 46], which found that comorbid perfectionistic tendencies in adulthood were higher in AN individuals with a lower BMI (i.e., severe AN group) than those with a higher BMI (i.e., mild severity AN group). Contrastingly, a recent study by Krug et al. [24] did not find significant differences in adult clinical perfectionism across both the DSM-5 BMI and DT severity groups among individuals with AN. These discrepancies in findings may be either due to the heterogeneous samples across these studies or may have been confounded by the level of care obtained by individuals across these studies. For instance, Dakanalis et al. [45] assessed an inpatient clinical AN sample, while both Krug et al. [24] and the current study comprised a mixture of inpatient, outpatient, and community participants. Another thing to consider is that the current study assessed childhood and not adulthood perfectionism, despite perfectionism being a stable trait [47], and childhood perfectionism is strongly associated with adulthood perfectionism [48].

No significant differences between either the DSM-5 BMI or DT AN severity groups in the rates of being exposed to the remaining significant risk factors were revealed in our follow-up analyses. Of interest, no significant differences in the frequency of childhood obesity across both the DSM-5 BMI and DT AN severity groups were found. Such findings are unexpected because in the current study childhood obesity was the strongest significant predictor for a subsequent AN diagnosis (OR ranges from 17 to 25). Furthermore, studies have consistently found that individuals with AN with premorbid obesity presented with significantly more severe physical (e.g., cardiovascular failure) and psychological sequelae, and poorer prognosis compared to AN individuals with premorbid normal weight [49, 50]. The current non-significant findings might be attributable to a small sample size across the three DSM-5 BMI and the two DT groups, lacking sufficient power to detect differences across severity groups.

Only Dang et al. [4] and Krug et al. [24] have compared the DSM-5 with the DT severity ratings for all EDs. Findings from both studies support the clinical utility of the DT severity rating over the DSM-5 in indexing ED and general psychopathology across most ED subtypes. In the current study, our regression analyses, however, did not reveal any significant association between the evaluated risk factors and the severity of AN, as determined by either the DSM-5 BMI or DT ratings. Previous studies [5, 22, 24] examined the clinical validity (i.e., significant differences in the level of symptomatology across severity groups), whereas the current study focused on the retrospective validity (i.e., ability to distinguish risk factors that are related to the development of different AN severity levels) of these severity ratings. Therefore, the DT severity rating for AN might only be superior to the DSM-5 BMI severity rating in distinguishing the level of AN-related concurrent symptomatology [4, 24], but may not be as effective in capturing retrospectively assessed risk factors. Nevertheless, this study was the first to examine the relationship between the DSM-5 BMI and DT severity indices and AN risk factors, hence replication of these results is needed.

### Implications

The staging model by Treasure et al. [51] can be applied to develop prevention and early treatment programs for AN. Based on our findings such an approach should use two steps. First, it would be important to identify individuals at risk of developing AN where significant AN risk factors (i.e., as per the current results, childhood obesity, being teased about weight, shape, and appearance, and feeling embarrassed about breasts) occur. It might be beneficial for these risk factors to be included in prevention programs for AN across all severity spectrums. As shown to be beneficial in previous research [52], cognitive dissonance-based interventions could aim to increase individuals’ appreciation of different body types, reducing pressure to be thin, and body dissatisfaction.

Second, we found that individuals with AN who exhibited perfectionistic traits during childhood were more likely to develop extreme severe AN per the DSM-5 BMI severity rating in adulthood. Such findings, therefore, underscore the importance of incorporating perfectionism in early prevention programs to prevent this trait from intensifying throughout adolescence and adulthood. While it might be advantageous to focus on perfectionism across all AN severity levels (e.g., identifying perfectionism triggers), implementing more comprehensive and targeted early perfectionism interventions (e.g., cognitive remediation therapy [CRT]; [53] might be highly beneficial for individuals who are at risk of developing extreme severe AN. Accordingly, CRT has demonstrated promising results in improving ED symptomatology among individuals with severe and enduring AN [54, 55]. Given the novelty of our current results, future research is warranted to explore the efficacy of additional perfectionism interventions, including CRT modules, in mitigating the progression of AN severity.

Finally, except for perfectionism, we found no other risk factors linked to varying DSM-5 AN severity levels, once again casting doubt on the validity of the proposed BMI-based severity rating by the DSM-5. While recognising the importance of severity ratings in prevention strategies and treatment planning, relying solely on BMI may oversimplify the complex nature of AN, encompassing elements such as fear of weight gain and body distortions [56]. This critique does not dismiss the use of a severity index for AN but underscores the necessity for an alternative perspective based on empirical data. Subsequent research should explore different indicators, such as duration of illness or perfectionism, to refine severity assessments, hence enhancing the efficacy of prevention and treatment strategies. Notably, shorter durations of illness have been proposed as a valuable severity indicator for AN [57, 58], correlating with higher BMI [57], more favourable treatment outcomes [58, 59], lower levels of perfectionism [60], and increased cognitive flexibility [61].

### Strengths and limitations

A strength of this research was our discordant case–control interview-based design to identify a wide range of psychosocial risk factors for AN. This means the current study was able to control for environmental factors which allowed to establish individual risk factors.

The current study also has a few limitations that need to be acknowledged. First, along with the exploratory nature of our research, we needed to categorise our difficult-to-recruit 153 AN sample into three DSM-5 BMI and two DT groups. This may not have provided sufficient power to detect a significant relationship between the assessed risk factors and the different DSM-5 severity levels. Future studies should employ a larger sample to ensure a more balanced distribution across the different DMS-5 BMI severity groups to gain a more accurate insight into the relationship between AN severity groups and risk factors.

Second, despite using the ORFI [11] to maximise reporting accuracy, potential recall biases are inherent to retrospective reporting. We did not include other informants in assisting with reporting family history but rather solely relying on the family history stated by the participants. Future research should include family members in the study process to bypass the inherent recall bias of retrospective assessment. Future studies should also consider a longitudinal design to assess the temporal relationship of the ORFI risk factors using different informants.

Third, our study departed from DSM-5 guidelines, categorising AN severity based on lowest BMI instead of current BMI. The lowest BMI was utilised because we recruited participants with a lifetime AN diagnosis, potentially including recovered and non-recovered cases. The inclusion of both groups poses a confounding factor, as non-recovered individuals might reach lower BMIs, leading to shifts between DSM-5 severity groups. Furthermore, the divergence between the DSM-5 severity groups based on lowest BMI and our DT severity groups, which were based on DT levels at the time of assessment, alongside with having a mixture of inpatient, outpatient, and community-based individuals with AN recruited across Europe might have confounded the results of the current study. Therefore, future studies should use the current BMI as per the DSM-5, instead of the lowest BMI, to examine the relationship between AN severity groups and risk factors in a sample of individuals with a current DSM-5 diagnosis of AN.

Fourth, our study exclusively included a female sample, while research has shown that different risk factors contribute to the development of AN in males and females [62]. As a result, the findings of the current study cannot be generalised beyond female-specific sample.

Lastly, in highlighting childhood perfectionism as a significant risk factor for extreme severe AN based on the DSM-5 severity rating, it is crucial to acknowledge a potential limitation in the scope of this finding. The DSM-5 AN severity level can shift from DSM-5 mild to DSM-5 extreme with just a one-point change in BMI. The DSM-5 extreme category, on the other hand, has a broad range (e.g., our participants' BMI in the extreme group had BMI ranged from 8.6 to 15 kg/m^2^), highlighting potential heterogeneity within the DSM-5 extreme severe group that requires a more detailed exploration for certainty about our study's implications. Future research should specify the DSM-5 extreme severe group into subgroups (e.g., very extreme severe DSM-5 group) for a detailed understanding of diverse characteristics and implications within the extreme severity spectrum.

## Conclusion

Our findings provided limited support for the DSM-5 BMI and DT AN severity ratings in identifying related risk factors for varying AN severity levels. However, BMI is an objective physical severity measure whereas DT is a cognitive measure of severity. In treatment, weight changes rapidly (especially in youth), but cognitive symptoms linger, highlighting the need to consider their distinct characteristics within a treatment context. Regardless, the current findings do highlight the significance of childhood perfectionism as a risk factor in developing extreme severe AN according to the DSM-5 BMI severity rating. It may be beneficial for prevention and early intervention programs to include a focus on managing perfectionism to reduce the severity of AN. As our study is the first of its kind, further replication, using several informants and longitudinal designs is needed to provide further insight into the relationship between risk factors for AN and DSM-5 BMI and other alternative severity ratings systems, including weight and shape concerns and, the number of purging methods.

### Supplementary Information

**Additional file 1**. Social and clinical related information of healthy sisters and individuals with AN.

## Data Availability

All data analysed during this study are included in this article (tables/figures). Raw data can be requested on reasonable requests.

## Abbreviations

AN: Anorexia nervosa
ANOVA: Analyses of variance
BED: Binge eating disorder
BMI: Body mass index
BN: Bulimia nervosa
CRT: Cognitive remediation therapy
DSM: Diagnostic statistical manual
DT: Drive for thinness
ED: Eating disorder
EDI-2: Eating disorder inventory-2
LIFE: Longitudinal interval follow-up evaluation
OR: Odd ratio
ORFI: Oxford risk factor interview
