# Supplementary material for: Do risk factors differentiate DSM-5 and drive for thinness severity groups for anorexia nervosa?
Source: J Eat Disord. 2024 Jan 11;12:5. doi: 10.1186/s40337-024-00966-5 (PMC10785425; doi:10.1186/s40337-024-00966-5)
Supplement: Supplementary file 1 — Additional file 1. Social and clinical related information of healthy sisters and individuals with AN. [file 40337_2024_966_MOESM1_ESM.docx]

**Supplementary 1**. Social and clinical related information of healthy sisters and individuals with AN

|  | | **Comparisons between healthy sisters and individuals with AN** | | | **Comparisons between DSM-5 severity groups** | | | | **Comparisons between Drive for thinness (DT) severity groups** | | |
| --- | --- | --- | --- | --- | --- | --- | --- | --- | --- | --- | --- |
| **Social information** | |  | | |  | | | |  | | |
|  | **Total**  **Mean(SD)** | **Healthy sisters** | **Individuals with AN** | **P values (V)** | **Mild/Moderate** | **Serve** | **Extreme Severe** | **P values (V)** | **Low DT** | **High DT** | **P values (V)** |
| **Participants’ employment status** | | | | .775 (.076) |  |  |  | .731 (.185) |  |  | .314 (.139) |
| Never employed | 4 (1.3%) | 2 (1.3%) | 2 (1.3%) |  | 1 (3.2%) | 0 (0.0%) | 1 (1.2%) |  | 1 (1.6%) | 0 (0.0%) |  |
| Employed previously | 32 (10.3%) | 16 (10.2%) | 16 (10.5%) |  | 3 (9.7%) | 5 (12.5%) | 8 (9.8%) |  | 3 (4.8%) | 12 (8.2%) |  |
| Employed | 136 (43.9%) | 74 (47.7%) | 62 (40.5%) |  | 16 (51.6%) | 15 (37.5%) | 31 (37.8%) |  | 25 (40.3%) | 61 (45.9%) |  |
| On social benefits | 8 (2.6%) | 3 (1.9%) | 5 (3.3%) |  | 0 (0.0%) | 1 (2.5%) | 4 (4.9%) |  | 2 (3.2%) | 2 (1.6%) |  |
| Student | 130 (41.9%) | 62 (39.5%) | 68 (44.4%) |  | 11 (35.5%) | 19 (47.5%) | 38 (46.3%) |  | 31 (50.0%) | 47 (44.3%) |  |
| **Mother’s education level** | | | | .997 (.012) |  |  |  | .033 (.212) |  |  | .657 (.081) |
| Primary school | 91 (29.3%) | 46 (29.1%) | 45 (29.3%) |  | 13 (41.9%) | 10 (25.0%) | 22 (26.8%) |  | 31 (33.9%) | 18 (29.5%) |  |
| Professional degree | 76 (24.4%) | 38 (24.1%) | 38 (24.4%) |  | 7 (22.6%) | 16 (40.0%) | 15 (18.3%) |  | 14 (22.6%) | 18 (29.5%) |  |
| Secondary school | 93 (29.9%) | 48 (30.4%) | 45 (29.9%) |  | 7 (22.6%) | 6 (15.0%) | 31 (39.0%) |  | 17 (27.4%) | 16 (26.2%) |  |
| University degree | 51 (16.4%) | 26 (16.5%) | 25 (16.4%) |  | 4 (12.9%) | 8 (20.0%) | 13 (15.9%) |  | 10 (16.1%) | 9 (14.8%) |  |
| **Father’s education level** | | | | 996 (.015) |  |  |  | .006 (.245) |  |  | .533 (.095) |
| Primary school | 63 (20.5%) | 32 (20.5%) | 41 (20.5%) |  | 13 (41.9%) | 8 (20.5%) | 10 (12.3%) |  | 13 (21.3%) | 11 (18.3%) |  |
| Professional degree | 82 (26.7%) | 41 (26.3%) | 31 (27.2%) |  | 7 (22.6%) | 15 (38.5%) | 19 (23.5%) |  | 20 (32.8%) | 20 (33.3%) |  |
| Secondary school | 75 (24.4%) | 39 (25.0%) | 36 (23.8%) |  | 3 (9.7%) | 8 (20.5%) | 25 (30.9%) |  | 10 (16.4%) | 14 (23.3%) |  |
| University degree | 87 (28.3%) | 44 (28.2%) | 43 (28.5%) |  | 8 (25.8%) | 8 (20.5%) | 27 (33.3%) |  | 18 (29.5%) | 15 (25.0%) |  |
